# Development of an integrated risk stratification model for metastatic medulloblastoma (M2/3) using clinical, radiologic, and molecular variables

**DOI:** 10.1093/noajnl/vdaf265

**Published:** 2025-12-22

**Authors:** Wen-Tao Zhou, Tao Wu, Yu-Fei Lu, Shu-Xu Du, Han-Guang Zhao, Si-Kang Ren, Chi Zhao, Yong-Ji Tian, Fu Zhao

**Affiliations:** Department of Cell Biology, Beijing Neurosurgical Institute, Capital Medical University, Beijing, China; Department of Neurosurgery, Beijing Tiantan Hospital, Capital Medical University, Beijing, China; Neurosurgical Department, Beijing Friendship Hospital, Capital Medical University, Beijing, China; The Eighth Clinical School, Fuxing Hospital, Capital Medical University, Beijing, China; Pediatric Department, Beijing Shijitan Hospital, Capital Medical University, Beijing, China; Department of Pediatric Neurosurgery, Beijing Neurosurgical Institute, Capital Medical University, Beijing, China; Department of Pediatric Neurosurgery, Beijing Neurosurgical Institute, Capital Medical University, Beijing, China; Department of Neuro-Oncology, Sanbo Brain Hospital, Capital Medical University, Beijing, China; Department of Neurosurgery, Beijing Tiantan Hospital, Capital Medical University, Beijing, China; Department of Pediatric Neurosurgery, Beijing Neurosurgical Institute, Capital Medical University, Beijing, China; Department of Pediatric Neurosurgery, Beijing Neurosurgical Institute, Capital Medical University, Beijing, China

**Keywords:** adjuvant therapy, medulloblastoma, metastasis, molecular subgroup, prognostic factors

## Abstract

**Background:**

Approximately 20% of medulloblastoma (MB) patients are diagnosed with metastatic disease and typically exhibit extremely poor clinical outcomes. This study aimed to investigate potential prognostic factors affecting the survival of patients with metastatic MB.

**Methods:**

Patients with initial diagnosis of metastatic MB (M2/3) at Beijing Tiantan Hospital were included. Radiological characteristics were discerned through a retrospective review. Overall survival (OS) and event-free survival (EFS) were calculated using the Kaplan-Meier analysis. Multivariable Cox proportional hazards model was employed to identify potential prognostic factors.

**Results:**

This study included 115 patients with M2/3 MB. Group 4 MBs accounted for 59.1% of cases (68/115). Kaplan-Meier analysis indicated that the 5-year EFS and OS rates were 56.1% and 68.7%, respectively. Patients with metastatic Group 3 MB and spinal metastases exhibited dismal outcomes. Postoperative “sandwich” strategy significantly prolonged 5-year EFS and OS rates. Multivariate COX regression models demonstrated that molecular subgrouping, metastatic patterns, and “sandwich” strategy were independent prognostic predictors for both EFS and OS in patients with M2/3 MB.

**Conclusion:**

Our study provides a novel risk stratification model for metastatic medulloblastoma that could potentially facilitate the development of individualized therapeutic strategies for the very high-risk patient population.

Key Points

- A new risk model aids treatment decisions in metastatic medulloblastoma.
- Molecular subgrouping is key to prognosis in M2/3 medulloblastoma patients.
- Postoperative “sandwich” strategy improves survival in M2/3 medulloblastoma.

Importance of the Study

Metastatic medulloblastoma (MB) (M2/3) carries poor outcomes, with limited prognostic models available. This study represents one of the largest single-center cohorts of metastatic medulloblastoma to date. We demonstrate that molecular subgroup, spinal dissemination, and use of postoperative “sandwich” therapy are independent predictors of survival. Patients with Group 3 MB or spinal metastasis showed poor outcomes, while the sandwich strategy significantly improved prognosis. These findings enhance current risk stratification and support individualized treatment approaches for high-risk pediatric MB, offering valuable real-world evidence to inform future clinical decision-making and trial design.

Medulloblastoma (MB) is the most common malignant pediatric brain tumor, known for its propensity to infiltrate and metastasize within the spinal and intracranial regions.^1^^,^^2^ Metastatic disease at the time of initial diagnosis is observed in over 20% of pediatric patients with MBs.^3^^,^^4^ These patients often face poor outcomes, with both the 5-year overall survival (OS) and event-free survival (EFS) rates falling below 50%.^5-7^ There remains substantial heterogeneity in treatment approaches, and a unified, prospectively validated standard specifically for ­radiographically metastatic (M2/3) disease has not been established.^8^^,^^9^ Though the intensification of radiotherapy and additional cycles of maintenance chemotherapy may improve outcomes, this approach could potentially result in devastating neurocognitive sequelae.^10-12^ Therefore, individualized treatment approaches are urgently required to further increase survival and improve quality of life in patients with metastatic MB.

The current consensus has identified four principal molecular subgroups of MB, named Wingless (WNT), Sonic Hedgehog (SHH), Group 3 (G3), and Group 4 (G4), with distinctive clinicopathologic and molecular features.^13^^,^^14^ Recent studies have demonstrated that G3 and G4 MBs have higher rates of metastatic dissemination compared to other subgroups.^13^^,^^15^^,^^16^ Notably, subgroup-specific radiologic patterns have been described—G3 MBs are frequently characterized by laminar (leptomeningeal/diffuse) metastases, whereas G4 MBs more commonly present nodular metastases and suprasellar spread.^3^

The treatment of MB has evolved considerably over the past decade. Treatment intensity for patients with MB is now stratified using risk-associated clinical features and molecular subgrouping, with the aim of maximizing cure rates while minimizing long-term disease-related and therapy-associated adverse effects.^17^ Several risk-adapted and molecularly guided trials, such as SJMB12, SIOP-HR-MB, and EORTC 1634-BTG/NOA-23, are currently being evaluated to optimize clinical treatment strategies for MB across molecular subgroups.^18-20^ In these trials, low-risk patients received de-escalated treatment, whereas high-risk or metastatic patients are assigned to more intensive regimens. For high-risk patients, studies such as POG, SJMB03, and HART/HIT have combined higher-dose craniospinal irradiation (CSI) with intensive chemotherapy, which has improved survival in some patients.^21-24^ Despite these significant advances, a unified and prospectively validated standard of care for patients with metastatic (M2/3) disease remains to be established.

To address the gap, we conducted a prognostic analysis in a large single-institution cohort of patients with radiographically metastatic MB (M2/3), categorized by molecular subgroup and centrally reviewed for radiologic patterns. We demonstrated that radiologic features, molecular subgroups, and adjuvant treatment strategies are significantly associated with patient outcomes. Our findings establish a robust foundation for risk stratification and highlight the importance of individualized treatment strategies in this patient population.

## Methods

### Patient Selection

After obtaining approval from the institutional review board (KY 2021-027-03) and a waiver of informed consent, we included patients with a confirmed diagnosis of metastatic MB at Beijing Tiantan Hospital between January 2009 and December 2021 in this study. Complete preoperative and postoperative enhanced MR images, as well as continuous follow-up data, were collected. The metastatic stage was determined based on the preoperative MR imaging according to Chang’s classification.^25^ Patients with metastatic (M2/M3) MB were included in this study. Patients lacking postoperative treatment records or with incomplete follow-up data were excluded from this study.

### Radiological Analysis

Diagnostic MR images obtained before surgery were independently evaluated by neurosurgeons/neuroradiologists who were blinded to the molecular subgrouping information. For discordant data between the two observers, a third senior neuroradiologist would review the case, and a consensus would be reached among the three observers at the end. The number of metastases was classified into single metastasis and multiple metastases. A single metastasis was defined by the presence of measurable lesions. The location of initial metastasis was categorized into three groups: supratentorial (ST), posterior fossa (PF), and spinal. Metastases located in either ST or PF or both, were classified as intracranial metastases, while those involving the spine—regardless of intracranial involvement—were designated as spinal metastases. The pattern of the initial metastases was classified as either nodular or laminar metastases. Nodular metastases were defined as round or ovoid lesions with measurable dimensions on contrast-enhanced MRI. Laminar metastases were characterized by linear leptomeningeal enhancement without a dominant discrete nodule; cases with extensive (“diffuse”) leptomeningeal enhancement were also classified as laminar. The distinction between single and multiple lesions was applied exclusively to nodular disease, whereas laminar metastases were considered a distinct pattern and not quantified numerically. When both types are present, the classification prioritizes laminar metastases.^26^ The enhancement pattern of primary tumor and metastatic lesions was categorized as either complete enhancement (≥90%) or partial/none enhancement (<90%).^27^

### Surgical Treatment

All patients underwent surgical treatment for primary tumor resection before adjunctive therapy. Gross total resection (GTR) was defined as no residual primary tumor based on post-operative enhanced MRI. Near-total resection (NTR) was defined as residual tumor burden of less than 1.5 cm.^24^

### Pathological and Molecular Analyses

Hematoxylin-eosin-stained slides of formalin-fixed paraffin-embedded material of the primary tumor samples were available for histopathological diagnosis and molecular classification.^28^ The NanoString system (NanoString Technologies, Seattle, Washington) or RNA sequencing was employed for determining the four molecular subgroups of MB, as described previously.^29^

### Adjuvant Therapies

The adjunctive therapy strategy employed an intensified “sandwich” strategy based on the clinical trial HIT 2000,^11^ comprising 1-2 cycles (each cycle lasting 2-3 weeks) of cyclophosphamide in combination with vincristine, high-dose methotrexate, carboplatin, etoposide, and intraventricular/intrathecal administration of methotrexate for intensification. CSI was followed with a total dose of 36 Gy to the whole brain and spinal cord, and 54 Gy to the posterior fossa or metastatic lesions. After that, patients continued with 8-10 cycles of maintenance chemotherapy, including vincristine, cisplatin, and lomustine.^11^ Patients under 3 years of age only received chemotherapy. CSI and maintenance chemotherapy will be administered once they reach the age of 3 years. Patients who did not complete the full treatment sequence were classified as “non-sandwich” group: (i) children younger than 3 years who could not receive CSI; (ii) patients who required radiotherapy due to rapid early disease progression; and (iii) individuals who discontinued the planned treatment region due to social reasons. MRI scans were performed following induction chemotherapy and radiotherapy, respectively, to evaluate treatment response. Radiographic response was defined as a ≥50% reduction in measurable tumor or metastatic lesion size compared with the postoperative baseline.^21^

### Statistical Analysis

Categorical variables were presented as counts and percentages (%), and continuous variables were expressed as the mean ± standard deviation. The t-test was utilized to evaluate differences between continuous variables. Fisher’s exact tests were used to assess the association between categorical variables. EFS and OS were defined as the time from initial tumor resection to date of first progression or relapse (EFS), or to date of death or last follow-up visit (OS). Five-year EFS and OS were estimated using the Kaplan-Meier method, with *P* values calculated via the log-rank test. Cox regression models with backward stepwise selection (inclusion criterion: univariate analysis, *P* value <.1) were used to analyze the prognostic impact of clinical, radiographic and histopathological factors. A nomogram was developed using the “rms” package in R (version 4.3.1) based on the Cox model to predict 1-, 3-, and 5-year EFS and OS rates. Calibration curves were generated to compare the associations between observed and predicted outcomes. Time-dependent receiver operating characteristic (ROC) curve analysis was used to evaluate the discriminative ability of the nomogram (“time ROC” package in R). Statistical analyses were performed using the R statistical environment (v4.3.1). Statistical significance was set at *P *< .05.

## Results

### Patient Characteristics

This study included 115 patients (105 children and 10 adults) with metastatic MB. The mean age of all patients was 9.5 years (range: 2.5-49.5 years). The female-to-male ratio was 2.8:1. During operation, GTR for primary tumor was achieved in 49.6% (57/115) of patients, while NTR was performed in 50.4% (58/115) of patients due to tumor adhesion with the brainstem. No intracranial hemorrhage or severe infection, leakage of cerebrospinal fluid or death, were observed postoperatively. Pathological analysis revealed 101 classic medulloblastomas (CMBs) (87.8%), 7 desmoplastic/nodular medulloblastomas (DNMBs) (6.1%), and 7 large cell/anaplastic medulloblastomas (LC/AMBs) (6.1%). Molecular subgrouping analysis identified 6 WNT MBs (5.2%), 16 SHH MBs (13.9%), 25 G3 MBs (21.7%), and 68 G4 MBs (59.1%). Immunohistochemical staining showed p53 negativity in all cases. The details are provided in Table 1.

**Table 1.** Characteristics of metastatic medulloblastoma and clinical features of patients

Patient characteristics	Number of cases (%) or mean (range)
No. of patients	115 (100)
Gender	
Male	30 (26.1)
Female	85 (73.9)
Age (y)	9.5 ± 7.6 (2.5-49.5)
≥18	10 (8.7)
3-18	101 (87.8)
<3	4 (3.5)
Primary tumor size (mm)	
<35	24 (20.9)
≥35	91 (79.1)
Primary tumor enhancement	
Partial/none	95 (82.6)
Complete	20 (17.4)
Cyst	
Yes	43 (37.4)
No	72 (62.6)
Primary tumor location	
Midline	96 (83.5)
Lateral	19 (16.5)
Extent of surgery	
NTR	58 (50.4)
GTR	57 (49.6)
Histology	
CMB	101 (87.8)
DNMB	7 (6.1)
LC/AMB	7 (6.1)
Molecular subgroups	
WNT	6 (5.2)
SHH	16 (13.9)
Group 3	25 (21.7)
Group 4	68 (59.1)
Location of metastasis	
Intracranial	95 (82.6)
Spinal	20 (17.4)
Number of metastases	
Single	47 (40.9)
Multiple	68 (59.1)
Pattern of metastasis	
Nodular	74 (64.3)
Laminar	41 (35.7)
Enhancement of metastasis	
Partial/none	27 (23.5)
Complete	88 (76.5)
Adjuvant treatment	
Sandwich	88 (76.5)
Non-sandwich	27 (23.5)
EFS (mo)	40.9 ± 34 (3-126)
OS (mo)	47.3 ± 27.9 (4-126)
Progression	
Yes	65 (56.5)
No	50 (43.5)
Survival	
Alive	84 (73.0)
Dead	31 (27.0)

Abbreviations: CMB, classic type medulloblastoma; DNMB, desmoplastic/nodular type medulloblastoma; EFS, event-free survival; GTR, gross total resection; LC/AMB, large cell/anaplastic medulloblastoma; MBEN, medulloblastoma with extensive nodularity; NTR, near total resection; OS, overall survival.

### Radiological Features

**Figure 1.**
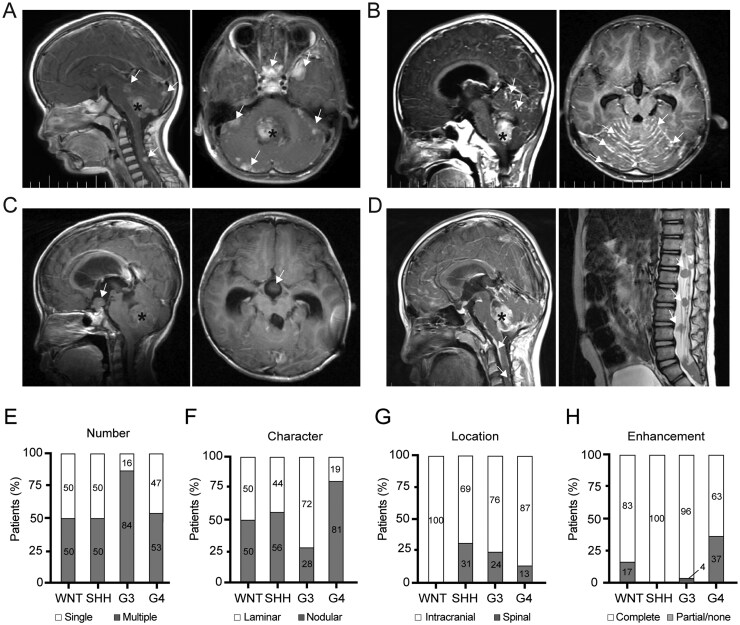
Axial and sagittal T1-weighted MR images of medulloblastoma metastasis. (A) Representing MR images showing a case of G3 MB with multiple nodular metastases present in the suprasellar region, left temporal lobe and bilateral cerebellar hemispheres. The asterisk indicates the primary tumor, while the white arrows mark the metastatic lesions. (B) Representing MR images showing a case of G3 MB with laminar metastases in the bilateral cerebellar hemispheres. (C) Representing MR images showing a case of G4 MB with the minimal/no enhancement of the primary tumor and a nodular lesion in the suprasellar region. (D) Representing MR images of a G3 MB showing laminar lesions in the cervical spinal cord and nodular metastases in the lumbar spinal cord. (E-H) Stacked bar plots illustrating the subgroup-specific metastatic patterns across the four molecular subgroups of MB.

Radiological analysis showed that single metastasis was found in 40.9% of cases (47/115), whereas multiple metastases were identified in 59.1% (68/115) (Figure 1A). Nodular and laminar metastases were identified in 64.3% and 35.7% of cases, respectively (Figure 1B and C). Partial/none enhancement was found in 23.5% (27/115) of metastases (Figure 1C). Intracranial metastases were observed in 82.6% of cases (95/115), and spinal metastases were found in 17.4% of cases (20/115) (Figure 1D; Table 1).

Among four molecular subgroups of MBs, G3 MBs exhibited a significantly higher proportion of multiple metastases compared to other subgroups (*P *= .041; Figure 1E; Table S1). G4 MBs showed greater prevalence of nodular metastases, whereas G3 MBs displayed a higher incidence of laminar metastases (*P *< .001; Figure 1F). Metastases of G4 MBs predominantly located in the intracranial region compared to other subgroups (*P *= .006), and no spinal metastases were observed in WNT MBs (Figure 1G; Table S1). Additionally, a higher portion of partial/none enhancement was noted in metastatic G4 MBs (*P *= .006) (Figure 1H).

### Prognosis

Postoperatively, 76.5% (88/115) of patients received intensified “sandwich” strategy. Twenty-seven patients did not receive the complete sandwich treatment cycle for various reasons: 19 patients received only radiotherapy or radiotherapy combined with maintenance chemotherapy due to social factors; one patient with early progressive disease; seven patients received postoperative chemotherapy as they were under 3 years of age. Toxicity of maintenance chemotherapy, including neutropenia, neurotoxicity and ototoxicity was observed in 21 patients. Therapy delays (>1 week) or modifications were required in 17 patients. No fatal adverse drug reactions were observed. In the sandwich treatment cohort, 90% of patients (79/88) achieved a radiographic response after induction chemotherapy, and 95% (84/88) after radiotherapy. Tumor progression was observed in all patients who did not achieve a radiographic response, and 80% of these patients died during follow-up.

The mean follow-up period was 77.0 months (range: 28.0-126.0 months). During follow-up, tumor progression or recurrence was observed in 43.5% (50/115) of patients. The mean time from tumor resection to tumor recurrence was 40.9 months (range 3.0-126.0 months). Twenty-seven percent of patients (31/115) died by the last follow-up. The estimated 5-year EFS and OS rates were 56.1% (95% CI: 54.5-57.6) and 68.7% (95% CI: 67.0-71.8), respectively.

**Figure 2.**
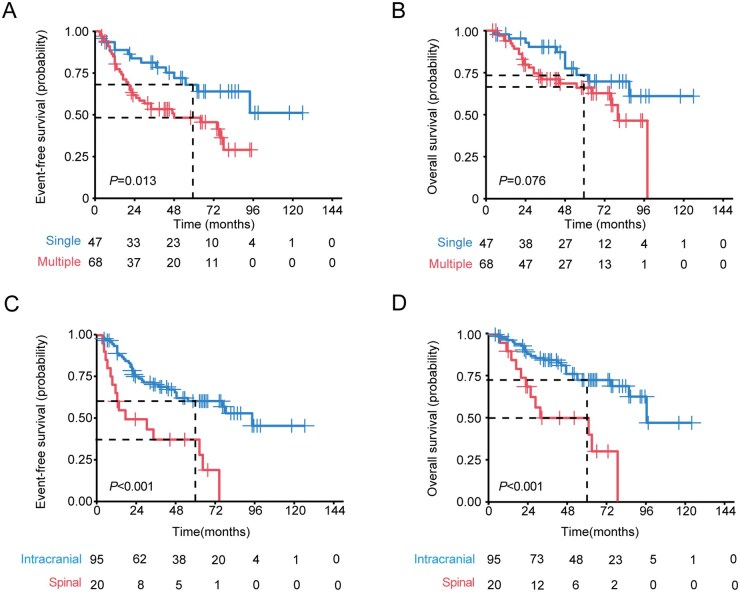
Kaplan-Meier plots illustrating the estimated event-free survival and overall survival time distributions, stratified by the number (A and B) and the location (C and D) of initial metastases.

Kaplan-Meier survival analysis revealed that patients with a single metastatic lesion had better 5-year EFS than those with multiple metastatic lesions (single: 68.0% [95% CI: 54.1-85.5], multiple: 48.3% [95% CI: 36.9-63.2]; *P *= .013) (Figure 2A), while no significant difference in OS was observed (Figure 2B). Patients with spinal metastasis (EFS: 36.8% [95% CI: 20.1-67.4], OS: 50.1% [95% CI: 31.2-80.2]) showed the poorer outcomes compared to those with intracranial metastases (EFS: 60.1% [95% CI: 49.9-72.4], OS: 72.8% [95% CI: 62.9-84.1]) (Figure 2C and D). No significant differences in OS or EFS across the three age categories (Figure S1).

**Figure 3.**
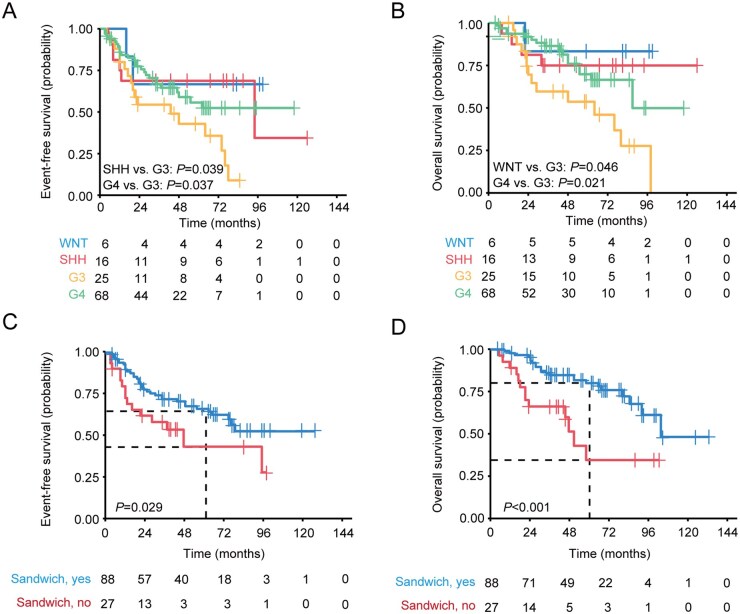
Kaplan-Meier plots illustrating the estimated event-free survival and overall survival time distributions, stratified by the molecular subgrouping (A and B) and adjuvant therapy (C and D).

Among four molecular subgroups, metastatic G3 MBs (5-year EFS: 42.9% [95% CI: 26.1-70.4]; 5-year OS: 53.7% [95% CI: 35.9-80.3]) displayed dismal outcomes compared with the other subgroups (5-year EFS: SHH, 68.8% [95% CI: 49.4-95.7]; G4, 55.7% [95% CI: 43.1-71.9]; 5-year OS: WNT, 83.3% [95% CI: 58.2-100.0]; G4, 69.9% [95% CI: 57.2-80.3]) (Figure 3A and B). Moreover, “sandwich” strategy significantly prolonged both 5-year EFS and OS in patients with metastatic MB (5-year EFS: sandwich, 61.2% [95% CI: 51.0-73.4]; non-sandwich, 35.2% [95% CI: 17.3-71.7]; 5-year OS: sandwich, 78.0% [95% CI: 68.9-88.4]; non-sandwich, 27.0% [95% CI: 11.5-67.9]) (Figure 3C and D).

### Univariate and Multivariate Analyses

**Figure 4.**
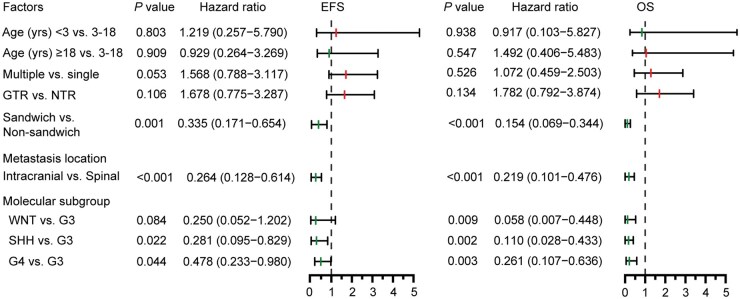
Association of clinical, radiologic, and histopathological factors with event-free survival (EFS) and overall survival (OS) based on ­multivariate Cox regression models. Abbreviations: GTR, gross total resection; NTR, near total resection.

Univariate analysis showed that metastasis number, metastasis locations, molecular subgroups, and “sandwich” strategy were significantly associated with outcomes in patients with metastatic MB (Tables S2 and S3). Multivariate Cox regression analyses identified adjuvant therapy (sandwich vs. non-sandwich), location of metastases (intracranial vs. spinal), and molecular subgroup as independent prognostic factors in patients. For EFS, “sandwich” therapy was associated with better prognosis (HR = 0.335, 95% CI = 0.171-0.654, *P *= .001), and intracranial metastases were favorable compared to spinal metastases (HR = 0.264, 95% CI = 0.128-0.614, *P *< .001). For OS, “sandwich” therapy (HR = 0.154, 95% CI = 0.069-0.344, *P *< .001), intracranial metastases (HR = 0.219, 95% CI = 0.101-0.476, *P *< .001), and G3 MB (*P *< .01) were significantly associated with better outcomes (Figure 4; Tables S4 and S5).

### Nomogram Model

**Figure 5.**
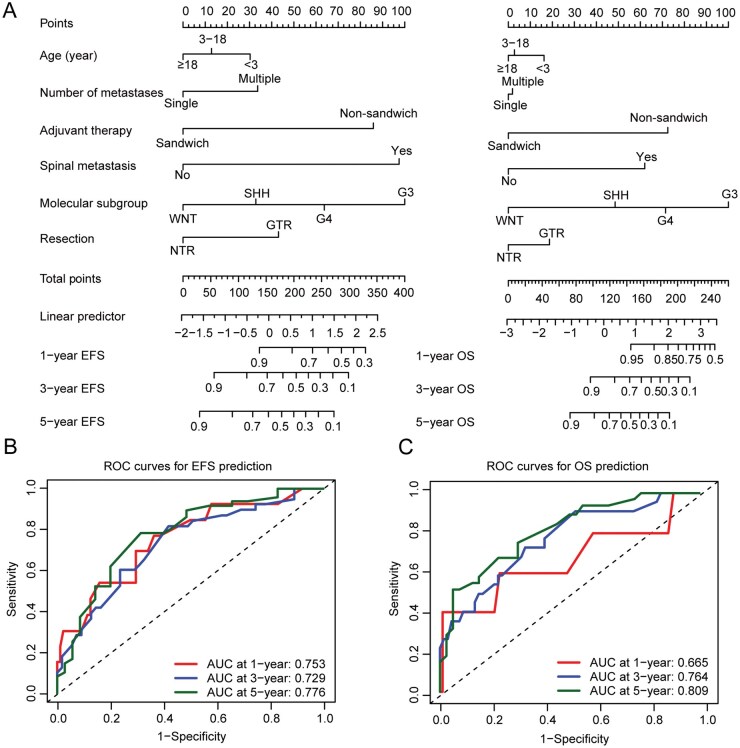
Nomogram models for prognostic prediction in patients with metastatic MB. (A) Nomograms were generated based on the multivariable Cox regression models. Each variable was translated into a risk score based on its contribution to the outcome of the model, and the scores for each case were then summed to calculate the total score. The sum of the scores from each variable was used to calculate the 1-, 3-, or 5-y EFS and OS probabilities. (B and C) Time-dependent ROC curves of 1-, 3-, and 5-y EFS (B) and OS (C) for predictive accuracy of nomograms. The prognostic predictive accuracies were analyzed using the area under the ROC curves (AUCs) at 1, 3, and 5 y. Abbreviations: EFS, event-free survival; GTR, gross total resection; NTR, near total resection; OS, overall survival; ROC, receiver operating characteristic.

Nomogram models were established to predict EFS and OS at 1, 3, and 5 years, based on multivariate Cox regression analysis (Figure 5A). Time-dependent ROC curves indicated good predictive accuracy for both EFS and OS, with area under the curve (AUC) values ranging from 0.729 to 0.776 for EFS and from 0.665 to 0.809 for OS (Figure 5B and C). In addition, the calibration plots demonstrated good consistency between nomogram-predicted probabilities and actual observations (Figure S2).

## Discussion

To date, the survival outcomes for patients with metastatic MB remain suboptimal (Table S6). Moreover, developing precise treatment strategies for these patients remains challenging due to the high progression and the relative rarity of this disease. Previous studies have demonstrated that spinal or laminar metastasis are associated with poor outcomes in this group patients.^26^^,^^30^^,^^31^ In a large single-institution cohort restricted to M2/3 disease, we systematically examined long-term outcomes in relation to radiologic features, molecular subgrouping, and adjuvant therapy. We found that spinal metastasis and G3 MB are two independent prognostic factors associated with poor outcomes. More importantly, our data confirmed that postoperative “sandwich” strategy was consistently associated with improved EFS and OS. Overall, our findings offer potential for more accurate risk stratification and individualized treatment strategies for M2/3 MB.

It is well established that GTR combined with CSI and chemotherapy can significantly improve outcomes for the majority of patients with MB, however, no uniform standard exists for those with overt metastasis. Several recent studies have demonstrated that intensified treatment strategies may encourage the survival rates of patients with metastatic MB.^11^^,^^23^^,^^26^^,^^32^ In this study, we provided evidence that “sandwich” strategy may be an effective treatment for patients with M2/3 disease, achieving notably high 5-year EFS rate of 61.2% and OS rate of 78.1%. This finding provides evidence that patients with M2/3 disease could potentially benefit from intensifying chemotherapy strategies. Interestingly, in contrast to patients with isolated M1 disease, who may benefit from immediate radiotherapy,^33^^,^^34^ no significant improvement in outcome was observed in patients with M2/3 disease.^11^^,^^32^^,^^35^ Our results and those of other studies highlight that postoperative immediate chemotherapy may play a crucial role in reducing disease burden and enhancing the efficiency of radiotherapy for patients with M2/3 disease.^9^^,^^11^

Our study reveals significant variations in metastatic patterns among the four molecular subgroups of MB. We observed that the majority of G3 MBs exhibited multiple small laminar metastases throughout the central nervous system. This finding aligns with previous observations,^3^^,^^36^ and highlights the significant propensity of G3 MB cells to disseminate to the leptomeninges. Although the underlying mechanisms of leptomeningeal dissemination remain poorly understood, it persists as an ominous prognostic sign and represents the ultimate challenge in the treatment of MBs.^37^ We found that patients with metastatic G3 MB (M2/3) had the poorest outcomes. Notably, all G3 MB patients with spinal metastases experienced progression and died during follow-up period. Therefore, novel approaches, such as chimeric antigen receptor T-cell therapy,^38^ are urgently required for this patient population, and further investigation into these strategies is essential to advance clinical outcomes.

In contrast, patients with metastatic G4 MB exhibit relatively favorable outcomes compared to those with metastatic G3 MB. Meanwhile, no significant differences in survival rates were observed between patients with metastatic G4 MB and those with metastatic WNT or SHH subgroups, though G4 MB accounts for the majority of metastatic cases (59.1%). These findings suggest that metastatic G4 MBs (M2/3) may have less aggressive behavior and demonstrate a favorable response to “sandwich” strategy. Consistent with previous reports,^3^^,^^36^ we found that nearly half of metastatic G4 MBs presented with single suprasellar lesions with minimal/absent enhancement, a pattern associated with superior survival in our analysis. The survival heterogeneity within metastatic G4 further supports risk-adapted intensification.

Several clinical trials, including St Jude/SIOP/ACNS and other ongoing studies, aim to calibrate treatment intensity and sequencing based on molecular classification.^18^^,^^19^ Our study adds M2/3-specific clinical–radiologic evidence—spinal dissemination, metastatic number, and early chemotherapy within a sandwich schedule—that can be used alongside these frameworks to refine risk allocation and practical decision-making in radiographically metastatic disease. The incremental value of GTR in the molecular era has been questioned by recent studies.^17^^,^^39^ In our multivariable models adjusted for subgroup, dissemination pattern/number, and adjuvant therapy, extent of resection was not an independent predictor, aligning with these updated observations (Table S6). Historically, POG trials comparing induction-first and RT-first sequences showed that upfront induction chemotherapy does not necessarily improve survival over immediate CSI. In the molecular era, SJMB03 further refined risk-adapted therapy by tailoring CSI dose and chemotherapy intensity/timing to molecular subgroup and risk level, while European HIT/HIIT regimens have used high-intensity, multi-agent induction, achieving high radiologic response rates but at the cost of greater acut.^21^^,^^22^^,^^24^ Thus, our data indicate that the sandwich strategy may represent an acceptable treatment option for some patients with M2/3 MB.

Our study has several limitations. As a retrospective study, it may be subject to selection and recall bias, and treatment heterogeneity. Several important prognostic genetic factors (eg, MYC/MYCN amplification) were not included in our COX regression models. In addition, the nomogram should be regarded as a developmental model, external validation in larger, multicenter cohorts will be necessary before routine clinical implementation. Future large-scale, prospective, multicenter studies incorporating these genetic biomarkers will be essential to validate our findings and to further refine the risk stratification for patients with M2/3 MB.

## Conclusion

In summary, our study refines the understanding of the clinical behavior of M2/3 MB and provides novel evidence supporting the necessity of risk‑stratification for these very high-risk patients.

## Supplementary Material

vdaf265_Supplementary_Data

## Data Availability

All dataset and material are available from the corresponding authors on reasonable request.
