# Supplementary material for: Development of an integrated risk stratification model for metastatic medulloblastoma (M2/3) using clinical, radiologic, and molecular variables
Source: Neurooncol Adv. 2025 Dec 22;8(1):vdaf265. doi: 10.1093/noajnl/vdaf265 (PMC12924635; doi:10.1093/noajnl/vdaf265)
Supplement: vdaf265_Supplementary_Data [file vdaf265_supplementary_data.docx]

# Supplementary Data

**Title:** Development of an Integrated Risk Stratification Model for Metastatic Medulloblastoma (M2/3) Using Clinical, Radiologic, and Molecular Variables

**Journal:** Neuro-Oncology Advances

**Authors:** Wen-Tao Zhou, Tao Wu, Yu-Fei Lu, Shu-Xu Du, Han-Guang Zhao, Si-Kang Ren, Chi Zhao, Yong-Ji Tian, Fu Zhao

**Corresponding authors:** Fu Zhao (Email: zhaofu@ccmu.edu.cn), Beijing Neurosurgical Institute, Capital Medical University. Yong-ji Tian (Email: tianyongji@bjtth.org), Beijing Tiantan Hospital, Capital Medical University.

**This file includes:**

Supplementary Figure 1–2

Supplementary Tables 1–6

**
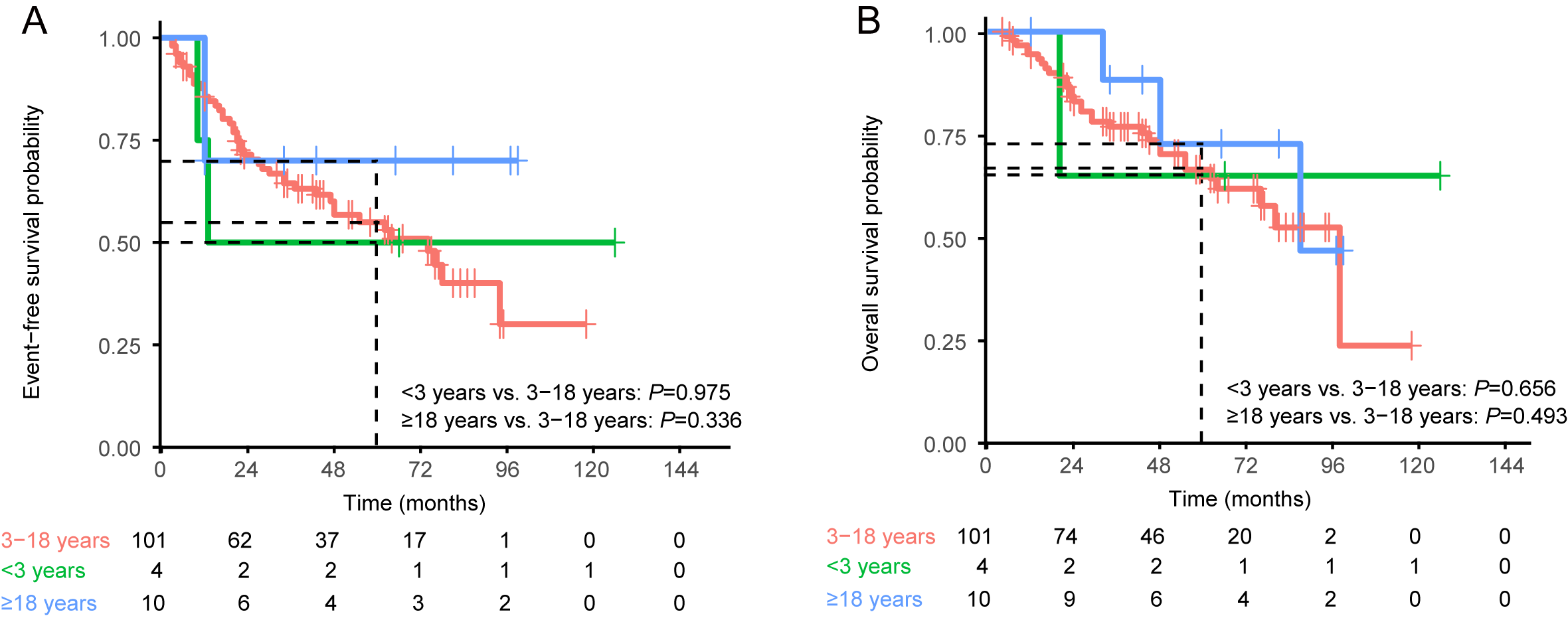
**

**Supplement Figure S1.** Kaplan–Meier curves for OS and EFS stratified by age group.


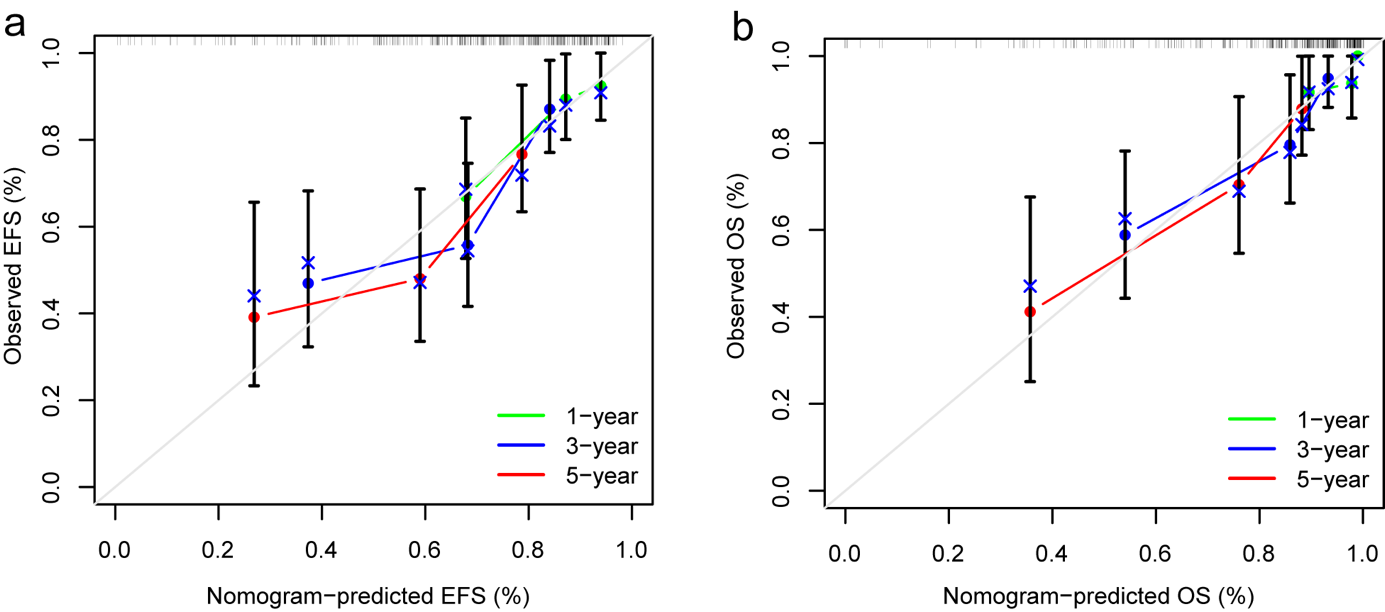


**Supplement Figure S2. a, b** Calibration curves of the nomograms for predicting (a) event-free survival (EFS) and (b) overall survival (OS) at 1, 3, and 5 years. The observed survival probabilities are plotted on the y-axis, and the nomogram-predicted probabilities are plotted on the x-axis.

**Supplementary Table S1.** Characteristics of metastases across four molecular subgroups of MB

| Variable | WNT | SHH | Group 3 | Group 4 | *P*^#^ |
| --- | --- | --- | --- | --- | --- |
| Metastases (number) |  |  |  |  | 0.041 |
| Single | 3 | 8 | 4 | 32 |  |
| Multiple | 3 | 8 | 21 | 36 |  |
| Metastases (character) |  |  |  |  | <0.001 |
| Nodular only | 3 | 9 | 7 | 55 |  |
| Laminar | 3 | 7 | 18 | 13 |  |
| Metastases (location)^*^ |  |  |  |  |  |
| Intracranial | 6 | 11 | 19 | 59 | <0.001 |
| Supratentorial | 4 | 6 | 9 | 53 | <0.001 |
| Infratentorial | 2 | 6 | 12 | 9 | 0.004 |
| Spinal | 0 | 5 | 6 | 9 | 0.173 |
| Metastases (enhancement) |  |  |  |  | 0.006 |
| Solid | 5 | 16 | 24 | 43 |  |
| Partial/none | 1 | 0 | 0 | 25 |  |

SP, supratentorial; Sup, suprasellar; PF, posterior fossa.

* The sum is > 115 because each case could have metastatic disease in 1-3 compartments.

# *P* value was calculated using χ^2^ test or Fisher exact test.

**Supplementary Table S2.** Association of factors with event free survival (EFS) based on univariate Cox regression analysis.

| Variables | EFS | | |
| --- | --- | --- | --- |
|  | HR | 95% CI | *P* |
| Sex, male vs. female | 1.019 | 0.541 − 1.919 | 0.955 |
| Age (years) |  |  |  |
| <3 vs. 3 − 18 | 1.011 | 0.243 − 4.183 | 0.992 |
| ≥18 vs.3 − 18 | 0.582 | 0.184 − 1.872 | 0.364 |
| Tumor size (cm), ≥35 vs. <35 | 1.069 | 0.557 − 2.053 | 0.841 |
| Metastasis number, multiple vs. single | 2.160 | 1.159 − 4.024 | 0.013 |
| Metastasis character, laminar vs. nodular | 0.915 | 0.513 − 1.632 | 0.763 |
| Extent of surgery, GTR vs. NTR | 1.666 | 0.944 − 2.941 | 0.075 |
| Metastasis location, intracranial vs. spinal | 0.311 | 0.168 − 0.576 | <0.001 |
| Adjuvant therapy, sandwich vs. non-sandwich | 0.514 | 0.279 – 0.947 | 0.033 |
| Molecular subgroup |  |  |  |
| WNT vs. Group 3 | 0.251 | 0.055 − 1.135 | 0.056 |
| SHH vs. Group 3 | 0.357 | 0.13 − 0.983 | 0.038 |
| Group 4 vs. Group 3 | 0.528 | 0.285 − 0.981 | 0.040 |
| Histology |  |  |  |
| CMB vs. DNMB | 1.174 | 0.604 − 2.285 | 0.635 |
| CMB vs. LC/AMB | 0.736 | 0.284 − 1.904 | 0.526 |
| Abbreviation: GTR, gross total resection; NTR, near total resection; CMB, classic medulloblastoma; DNMB, desmoplastic/nodular type medulloblastoma; LC/AMB, large cell / anaplastic medulloblastoma; EFS, event-free survival | | | |

**Supplementary Table S3.** Association of factors with overall survival (OS) based on univariate Cox regression analyses.

| Variables | OS | | |
| --- | --- | --- | --- |
|  | HR | 95% CI | P |
| Sex, male vs. female | 0.994 | 0.465 − 2.123 | 0.987 |
| Age (years) |  |  |  |
| <3 vs. 3 − 18 | 0.642 | 0.081 − 4.821 | 0.662 |
| ≥18 vs.3 − 18 | 0.681 | 0.203 − 2.242 | 0.521 |
| Tumor size, ≥35 vs. <35 | 1.045 | 0.485 − 2.253 | 0.911 |
| Metastasis number, multiple vs. single | 1.903 | 0.921 − 3.932 | 0.078 |
| Metastasis pattern, laminar vs. nodular | 1.313 | 0.572 − 2.228 | 0.721 |
| Extent of surgery, GTR vs. NTR | 1.365 | 0.699 − 2.665 | 0.361 |
| Metastasis location, intracranial vs. spinal | 0.182 | 0.068 − 0.486 | <0.001 |
| Adjuvant therapy, sandwich vs. non-sandwich | 0.253 | 0.096 − 0.669 | 0.003 |
| Molecular subgroups |  |  |  |
| WNT vs. Group 3 | 0.160 | 0.021 − 1.240 | 0.079 |
| SHH vs. Group 3 | 0.353 | 0.116 − 1.079 | 0.056 |
| Group 4 vs. Group 3 | 0.436 | 0.221 − 0.901 | 0.021 |
| Histology |  |  |  |
| CMB vs. DNMB | 1.126 | 0.520 − 2.438 | 0.763 |
| CMB vs. LCA/AMB | 1.195 | 0.349 − 4.085 | 0.776 |
| Abbreviation: GTR, gross total resection; NTR, near total resection; CMB, classic medulloblastoma; DNMB, desmoplastic/nodular type medulloblastoma; LC/AMB, large cell / anaplastic medulloblastoma; OS, overall survival | | | |

**Supplementary Table S4.** Association of factors with event − free survival (EFS) based on multivariate Cox regression analyses.

| Variables | EFS | | |
| --- | --- | --- | --- |
|  | HR | 95% CI | P |
| Age(years) |  |  |  |
| <3 vs. 3 − 18 | 1.219 | 0.257–5.790 | 0.803 |
| ≥18 vs.3 − 18 | 0.929 | 0.264–3.269 | 0.909 |
| Number of metastasis, multiple vs. single | 1.568 | 0.788 − 3.117 | 0.053 |
| Extent of surgery, GTR vs. NTR | 1.678 | 0.775 − 3.287 | 0.106 |
| Adjuvant therapy, sandwich vs. non-sandwich | 0.335 | 0.171 − 0.654 | 0.001 |
| Metastatic location, intracranial vs. spinal | 0.264 | 0.128 − 0.614 | <0.001 |
| Molecular subgroups |  |  |  |
| WNT vs. Group 3 | 0.250 | 0.052 − 1.202 | 0.084 |
| SHH vs. Group 3 | 0.281 | 0.095 − 0.829 | 0.022 |
| Group 4 vs. Group 3 | 0.478 | 0.233 − 0.980 | 0.044 |
| Abbreviation: GTR, gross total resection; NTR, near total resection; CMB, classic medulloblastoma; DNMB, desmoplastic/nodular type medulloblastoma; LC/AMB, large cell / anaplastic medulloblastoma; EFS, Event − free survival. | | | |

**Supplementary Table S5.** Association of factors with overall survival (OS) based on multivariate Cox regression analysis.

| Variables | OS | | |
| --- | --- | --- | --- |
|  | HR | 95% CI | *P* |
| Age (years) |  |  |  |
| <3 vs. 3 − 18 | 0.917 | 0.103–5.827 | 0.938 |
| ≥18 vs.3 − 18 | 1.492 | 0.406–5.483 | 0.547 |
| Metastasis number, multiple vs. single | 1.072 | 0.459 − 2.503 | 0.526 |
| Extent of surgery, GTR vs. NTR | 1.782 | 0.792 − 3.874 | 0.134 |
| Adjuvant therapy, sandwich vs. non-sandwich | 0.154 | 0.069 – 0.344 | <0.001 |
| Metastatic location, intracranial vs. spinal | 0.219 | 0.101 − 0.476 | <0.001 |
| Molecular subgroups |  |  |  |
| WNT vs. Group 3 | 0.058 | 0.007 − 0.448 | 0.009 |
| SHH vs. Group 3 | 0.110 | 0.028 − 0.443 | 0.002 |
| Group 4 vs. Group 3 | 0.261 | 0.107 − 0.636 | 0.003 |
| Abbreviation: GTR, gross total resection; NTR, near total resection; CMB, classic medulloblastoma; DNMB, desmoplastic/nodular type medulloblastoma; LC/AMB, large cell / anaplastic medulloblastoma; OS, overall survival | | | |

**Supplementary Table S6.** Literature review of metastatic medulloblastomas.

| Author and year | No. | Mean age (years） | Stage | Molecular subgroup | Metastasis location | Surgical resection | Adjuvant therapy | | 5-year EFS/OS |  |
| --- | --- | --- | --- | --- | --- | --- | --- | --- | --- | --- |
| Bouffet et al. 1994[23] | 23 | 7.5 | M1: 6  M2/3:17 | N/A | PF: 2  Spinal: 15 | GTR/STR: 17  PTR: 6 | | RT | EFS: 43.0% | |
| Meyers et al. 2000[22] | 37 | 9.4 | M3: 37 | N/A | Spinal: 37 | N/A | | RT+CT: 101  RT: 9  CT: 3 | OS: 24.0% | |
| Taylor et al. 2005[28] | 68 | 7.8 | M2/3: 68 | N/A | N/A | GTR: 17  STR: 42  biopsy: 7 | | CT: 7  CT+RT: 61 | EFS: 34.7%  OS: 43.9% | |
| Sanders et al. 2008[27] | 99 | 6.9 | M1: 18  M2/3: 81 | N/A | N/A | N/A | | N/A | EFS: 37.6% (M2/3)  OS: 51.1% (M2/3) | |
| Gandola et al. 2009[25] | 33 | 10 | M1: 9  M2: 6  M3: 17  M4: 1 | N/A | N/A | GTR: 17  STR: 15  biopsy: 1 | | CT:1  CT+HART:16  CT+HART+MC:16 | EFS: 70.0%  OS: 73.0% | |
| Dufour et al. 2012[18] | 117 | 4.0 | M1: 22  M2/3: 95 | N/A | N/A | N/A | | CT+CSI: 38  HDCT+ASCT+RT: 79 | EFS: 38.0%  OS: 45.0% | |
| Vivekanandan et al. 2015[24] | 34 | 7.0 | M1: 6  M2/3: 28 | N/A | N/A | N/A | | IC+RT+CT | EFS: 56.0%  OS: 56.0% | |
| Bueren et al. 2016[11] | 123 | 8.2 | M1: 36  M2/3: 87 | WNT: 4*  SHH: 4  G3: 20  G4: 41 | N/A | GTR: 66  STR: 58 | | CT+RT+MC | EFS: 62.0%  OS: 74.0% | |
| Zapotocky et al. 2017[3] | 40 | N/A | N/A | SHH: 5  G3: 16  G4: 19 | SP: 21  PF: 23  Spinal: 27 | N/A | | N/A | N/A | |
| Mbemba et al. 2018[29] | 34 | 7.3 | N/A | WNT: 1  SHH: 5  G3: 13  G4: 15 | PF: 13  SP: 4  Spinal: 7 | N/A | | N/A | N/A | |

Abbreviation: GTR, gross total resection; STR, subtotal resection; PTR, partial resection; EFS, event − free survival; OS, overall survival; AT, auxiliary therapy; CT, chemotherapy; RT, radiotherapy; CSI, craniospinal irradiation; HDCT, high dose chemotherapy; ASCT, autologous stem cell transplantation; IC, induction chemotherapy; MC, maintenance chemotherapy; SP, supratentorial; PF, posterior fossa.

* Sixty-nine (56%) cases were available for molecular subgrouping in this study.
